# PolyGR‐Containing Aggregates in Alzheimer's Disease: A Novel Proteinopathy Associated with Clinical Features of AD Patients

**DOI:** 10.1002/alz70855_105969

**Published:** 2025-12-24

**Authors:** Cemal Akmese, Rodrigo Francisco Tomas, Huong T. Phuong, Ana Mijares, Shu Guo, Logan R. Bell, Svitlana Yegorova, Stefanie K. Ng, Jennifer L. Phillips, Alexandra N. Melloni, Olga Pletnikova, H. Brent Clark, Juan C. Troncoso, Bradley T. Hyman, Laura P. W. Ranum, Stefan Prokop, Lien Nguyen

**Affiliations:** ^1^ University of Florida, College of Medicine, Gainesville, FL, USA; ^2^ University of Florida, Gainesville, FL, USA; ^3^ Massachusetts Alzheimer's Disease Research Center, Charlestown, MA, USA; ^4^ Johns Hopkins University School of Medicine, Baltimore, MD, USA; ^5^ University of Minnesota, Minneapolis, MN, USA; ^6^ University of Florida / Fixel Institute for Neurological Diseases, Gainesville, FL, USA

## Abstract

**Background:**

Alzheimer's disease (AD) is a progressive neurodegenerative disorder characterized by cognitive impairment and the accumulation of beta‐amyloid (Aβ) plaques and neurofibrillary hyperphosphorylated tau (pTau) tangles. While rare mutations in *APP, PSEN1*, and *PSEN2* cause familial AD, the mechanisms and genetic components of sporadic AD, which accounts for the majority of cases, remain largely unknown. Our recent study showed poly‐glycine‐arginine containing (polyGR+) aggregates accumulated in AD autopsy brain tissue. We also showed polyGR+ aggregate levels are associated with increased pTau levels. It has been shown that hypertension and head injuries were significantly associated with AD, although molecular mechanisms are unclear. Here we test a hypothesis that polyGR levels are associated with clinical histories of AD patients.

**Method:**

We obtained autopsy brain tissues from 133 AD cases and 30 age‐similar controls and categorized cases as late‐onset (LOAD) vs. early‐onset AD, as described in the literature. We performed immunohistochemical staining on sequential hippocampal sections for polyGR and polyGR+ aggregates were measured in the hippocampal regions. We collected demographic and clinical information from the patients, including vascular comorbidities (hypertension, ischemic events) and TBI. Statistical analyses were performed to study if polyGR aggregates link with clinical features or histories in AD cases.

**Result:**

Statistical analysis revealed no significant gender differences in polyGR staining. However, polyGR accumulation was significantly elevated in AD cases, showing a 9.4‐fold increase compared to controls (*p* < 0.0001). In late‐onset AD (LOAD), vascular and metabolic risk factors were associated with increased polyGR staining. Specifically, a history of traumatic brain injury (TBI) correlated with a 3.87‐fold increase (*p* = 0.0082), while hypertension and high cholesterol were linked to 2.98‐fold (*p* = 0.0069) and 3.42‐fold (*p* = 0.0135) increases, respectively. However, these risk factors did not significantly influence pTau staining between LOAD and control cases.

**Conclusion:**

Our findings highlight the role of vascular and metabolic risk factors, including TBI, hypertension, and hypercholesterolemia, in promoting polyGR accumulation in AD. Notably, polyGR accumulation appears to occur independently of pTau aggregation. These insights provide a deeper understanding of AD pathogenesis and suggest potential avenues for further investigation into the molecular mechanisms linking vascular and metabolic factors to polyGR pathology.

